# Neuroprotection and neuroregeneration triggered through the FUS-induced opening of the blood-brain barrier in a Parkinson’s mouse model

**DOI:** 10.1186/2050-5736-3-S1-O19

**Published:** 2015-06-30

**Authors:** Elisa Konofagou, Gesthimani Samiotaki, Shutao Wang, Vernice Jackson-Lewis, Serge Przedborski

**Affiliations:** 1 Columbia University, New York, NY, United States

## Background/introduction

Over the past decade, numerous small- and large-molecule products have been developed for treatment of neurodegenerative diseases with mixed success. When administered systemically *in vivo*, the blood-brain barrier (BBB) inhibits their delivery to the regions affected by those diseases. A successful drug delivery system requires transient, localized, and noninvasive targeting of a specific tissue region as provided by Focused Ultrasound (FUS). Neurturin (NTN) is a neurotrophic factor that has demonstrated to have neuroprotective and regenerative effects on dopaminergic neurons *in vivo* using invasive drug delivery methods. Utilizing recombinant adeno-associated virus (rAAV), therapeutic genes can be delivered to the brain for long-lasting treatments. In this study, we investigate the neuroprotective and neuroregenerative effects of non-invasively delivered rAAV-GDNF vectors and NTN in a PD mouse model, respectively.

## Methods

FUS at 1.5 MHz with in-house polydisperse microbubbles was performed. In the NTN study, NTN bioavailability and downstream signaling were detected and quantified through immunostaining for NTN, phosphorylated RET, ERK1/2 and CREB. To test for neuroprotection and neuroregeneration, the 1-methyl-4-phenyl-1,2,3,6-tetrahydropyridine (MPTP) mouse model was used with FUS before and after MPTP administration, respectively. In the rAAV study, the expression of GDNF after 4 weeks due to AAV transduction was assessed in MPTP mice using FUS-mediated AAV delivery prior to MPTP administration (for neuroprotection only).

## Results and conclusions

In the neurotrophic study, within the first hour of NTN administration, triggering of the signaling cascade was detected downstream to the neuronal nuclei. In the adenoviral study, the ratio of ipsilateral (FUS treated for FUS only and AAV+FUS groups) to the contralateral side showed significantly higher live neurons ratio from the AAV+FUS group (ANOVA, p=0.03) when compared to the control, AAV only and FUS only groups. These findings thus indicate the potential of the FUS method to mediate transport of proteins and mediate gene delivery through the blood-brain barrier for reversibility of the PD phenotype.

